# Habitat requirements of the Himalayan red panda (*Ailurus fulgens*) and threat analysis in Jigme Dorji National Park, Bhutan

**DOI:** 10.1002/ece3.6632

**Published:** 2020-08-05

**Authors:** Pema Dendup, Tatyana Humle, Damber Bista, Ugyen Penjor, Choki Lham, Jigme Gyeltshen

**Affiliations:** ^1^ Jigme Dorji National Park Department of Forests and Park Services Ministry of Agriculture and Forests Royal Government of Bhutan Gasa Bhutan; ^2^ Durrell Institute of Conservation and Ecology School of Anthropology and Conservation University of Kent Canterbury UK; ^3^ Wildlife Science Unit School of Agriculture and Food Sciences The University of Queensland Gatton Qld Australia; ^4^ Nature Conservation Division Department of Forests and Park Services Ministry of Agriculture and Forests Royal Government of Bhutan Thimphu Bhutan; ^5^ Wildlife Conservation Research Unit Department of Zoology The Racanati‐Kaplan Centre University of Oxford Tubney UK

**Keywords:** anthropogenic activities, Environment Impact Assessment, habitat disturbances, habitat use, red panda, water source

## Abstract

Understanding the influence of anthropogenic disturbances on species’ habitat use and distribution is critical to conservation managers in planning effective conservation strategies and mitigating the impact of development. Few studies have focused on the Himalayan red panda (*Ailurus fulgens*) in Bhutan. This study aimed to assess the habitat requirements and threats to this endangered species in the Khamaed subdistrict of the Jigme Dorji National Park, Bhutan. We employed a transect walk and plot‐sampling survey design across two seasons, that is, winter and spring. In total, we surveyed 84 × 50 m radius circular plots along 51 km of existing trails within a 25.4 km^2^ study area. At 500 m intervals, we established plots at random distances and direction from the trail. We recorded direct sightings (*n = *2) and indirect signs (*n = 14*), such as droppings and footprints as evidence of red panda presence within an altitudinal range of 2,414–3,618 m. We also noted 21 tree and 12 understory species within plots with red panda evidence; the dominant tree species was the Himalayan hemlock (*Tsuga dumosa*) and the Asian barberry (*Berberis asiatica*) as an understory species. Red panda presence showed a significant positive association with distance to water sources and fir forests. Plant disturbance and infrastructure, such as power transmission lines, were identified as prominent anthropogenic threats in the study area. Based on our findings, we recommend the development and implementation of local forest management plans, livestock intensification programs, and strict application of environmental impact assessment regulations to promote the conservation of the red panda in the region.

## Graphical abstract

Red panda occurrence was positively associated with fir forest and negatively associated with distance to the nearest stream. Unlike many other studied, red panda occurrence was not associated with the bamboo presence. Disturbances such as plant disturbances and infrastructure were identified as a prominent disturbance having influence over red panda habitat use in the study site. Oddly, red pandas had a positive association with the infrastructure presence.

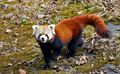

## 1 INTRODUCTION

Information and knowledge on species’ distribution are vital to understand their presence and infer habitat and ecological requirements (Elith & Leathwick, 2009; Noon, Bailey, Sisk, & McKelvey, 2012). Local small‐scale distribution surveys can shed light on species habitat preferences, existing threats, and responses to management interventions (Lahoz‐Monfort, Guillera‐Arroita, Milner‐Gulland, Young, & Nicholson, 2010). Such studies can help conservation managers plan and revisit species conservation policies and guide informed management decisions (Rayan & Linkie, 2015).

**Figure 1:**
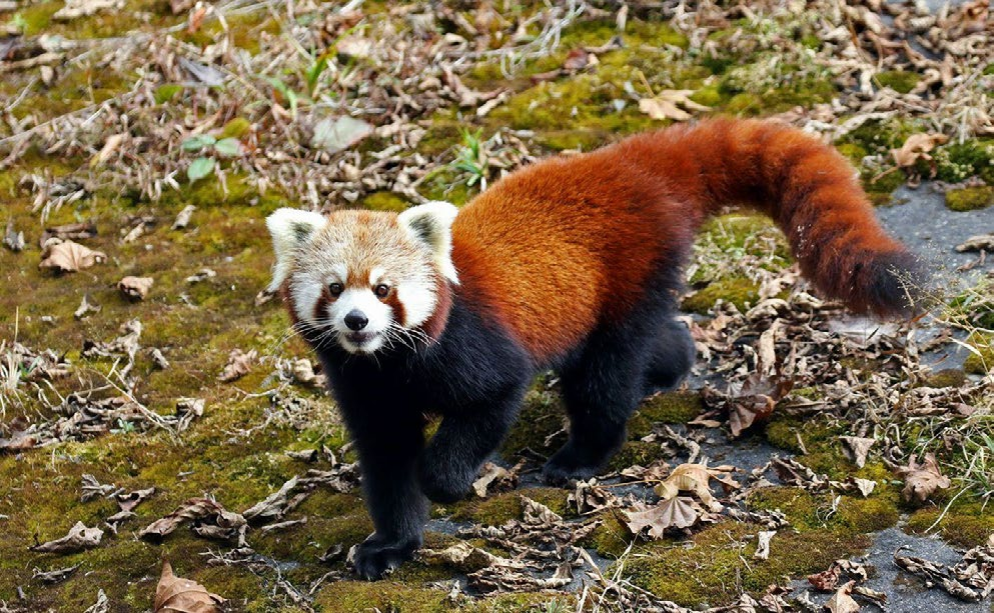
Red panda (*Ailurus fulgens*) photographed in Jigme Dorji National Park (Photograph: Sonam Dorji, JDNP)

The Red List of International Union for Conservation of Nature (IUCN) lists the red panda (Figure 1) as an endangered species (Glatston, Wei, Than, & Sherpa, 2015). Recently, Hu et al. (2020) recognized the red panda of Nepal, Bhutan, northern India, northern Myanmar, Tibet and western Yunnan Province of China as the Himalayan red panda (*Ailurus fulgens*), and its relative in Yunnan and Sichuan provinces of China as the Chinese red panda (*Ailurus styani*). This new taxonomic identification has underpinned the need of more studies to secure the survival of these two red panda species in the wild.

Globally, red pandas are reported from 49 protected areas (PAs) in China; 11 PAs in India; 10 PAs in Nepal; and 3 PAs in Myanmar (Thapa, Wu, et al., 2018). In Bhutan, red pandas are reported in eight PAs and six biological corridors (Dorji, Rajaratnam, & Vernes, 2012) and red panda areas in Bhutan account for about 43.5% of the predicted red panda habitat across range countries (Thapa, Wu, et al., 2018).

Red panda habitat, in general, includes evergreen forests, mixed broadleaf forests, deciduous forests, conifer mixed forests, and conifer forest with bamboo thicket understories (Wei, Feng, Wang, & Hu, 1999; Yonzon, Jones, & Fox, 1991). Bamboo leaves and shoots form the main bulk of the red panda diet with insects, grubs, lichens, and fruits acting as supplements (Choudhury, 1997; Yonzon & Hunter, 1991). Vegetation characteristics are strong predictors of habitat use (Ahlering et al., 2010). Studies have also indicated that red pandas favor habitats close to water sources (Bista et al., 2019; Pradhan, Saha, & Khan, 2001; Wei, Feng, Wang, & Hu, 2000; Yonzon & Hunter, 1991; Zhang, Hu, Han, & Wei, 2011; Zhou et al., 2013). However, habitat requirements for the red panda vary across different landscapes. In Phurmsingla National Park, habitats close to water sources were, for example, not a significant predictor of red panda presence (Dendup, Cheng, Lham, & Tenzin, 2016).

Threats to red panda survival are greatest in Bhutan, India, and Nepal than other range countries (Thapa, Hu, & Wei, 2018). Habitat loss, fragmentation, and degradation are some of the major threats to the red panda populations in these countries (Bista et al., 2017; Glatston et al., 2015; Pradhan et al., 2001; Wei et al., 1999; Yonzon & Hunter, 1991). The harvesting of forest resources and infrastructure development are some of the main drivers of red panda habitat destruction and fragmentation (Panthi, Khanal, Acharya, Aryal, & Srivathsa, 2017; Sharma & Belant, 2010; Sharma, Swenson, & Belant, 2014; Williams, 2003). Hickman, Roberts, and Larson (1993) reported habitat loss as a main cause for population decline, especially affecting endangered species that are sensitive to changes in their environment.

Red pandas are known to avoid areas close to human settlements and areas disturbed by livestock (Acharya et al., 2018; Dendup et al., 2016; Sharma et al., 2014; Wei et al., 2000). As habitat specialist, red pandas prefer less disturbed habitats; however, their responses to habitat disturbances may vary across locations (Acharya et al., 2018). Field observations made in some parts of Nepal have revealed extensive spatial overlap between the red panda and the livestock (Thapa et al., 2020). During winter months when ground vegetation is scarce, livestock are seen feeding on bamboo (Dendup et al., 2016; Sharma et al., 2014). As a generalist species, livestock adapt to feed on any available resources. Habitat‐generalist species in a community are reported to overexploit the environment and occupy habitats unexploited by habitat specialists at a larger spatial scale (Morris, 1996).

Poaching and illegal trade for pelts have been identified as important threats to the red panda, although their intensity varies across different countries (Badola, Fernandes, Marak, & Pilia, 2020; Bista, Baxter, & Murray, 2020; Xu & Guan, 2018). Free‐ranging domestic dogs also threaten red pandas and other wildlife. There are reports of stray dogs killing red pandas in Nepal and Bhutan (Bista & Paudel, 2014; Dorji et al., 2012; Williams, Dahal, & Subedi, 2011).

One of the major goals of conservation biology is to document environmental and anthropogenic factors that influence species’ distribution (Robinson, 2006). Understanding species’ habitat requirements and disturbances contributing to habitat destruction is very important to conserve endangered species (Hunt, Bayne, & Hache, 2017) like the red panda. Despite Jigme Dorji National Park (JDNP) harboring key red panda habitats in Bhutan (Dorji, Vernes, & Rajaratnam, 2011), very few studies have focused on documenting the conservation threats to this species in this region. This study hence aimed to fill this gap and assess the conservation status of the red panda in a critical but poorly studied area of the JDNP in Bhutan. In addition, this study sought to identify the threat factors affecting red panda habitat use.

## 2 MATERIALS AND METHODS

### 2.1 Study area

**Figure 2:**
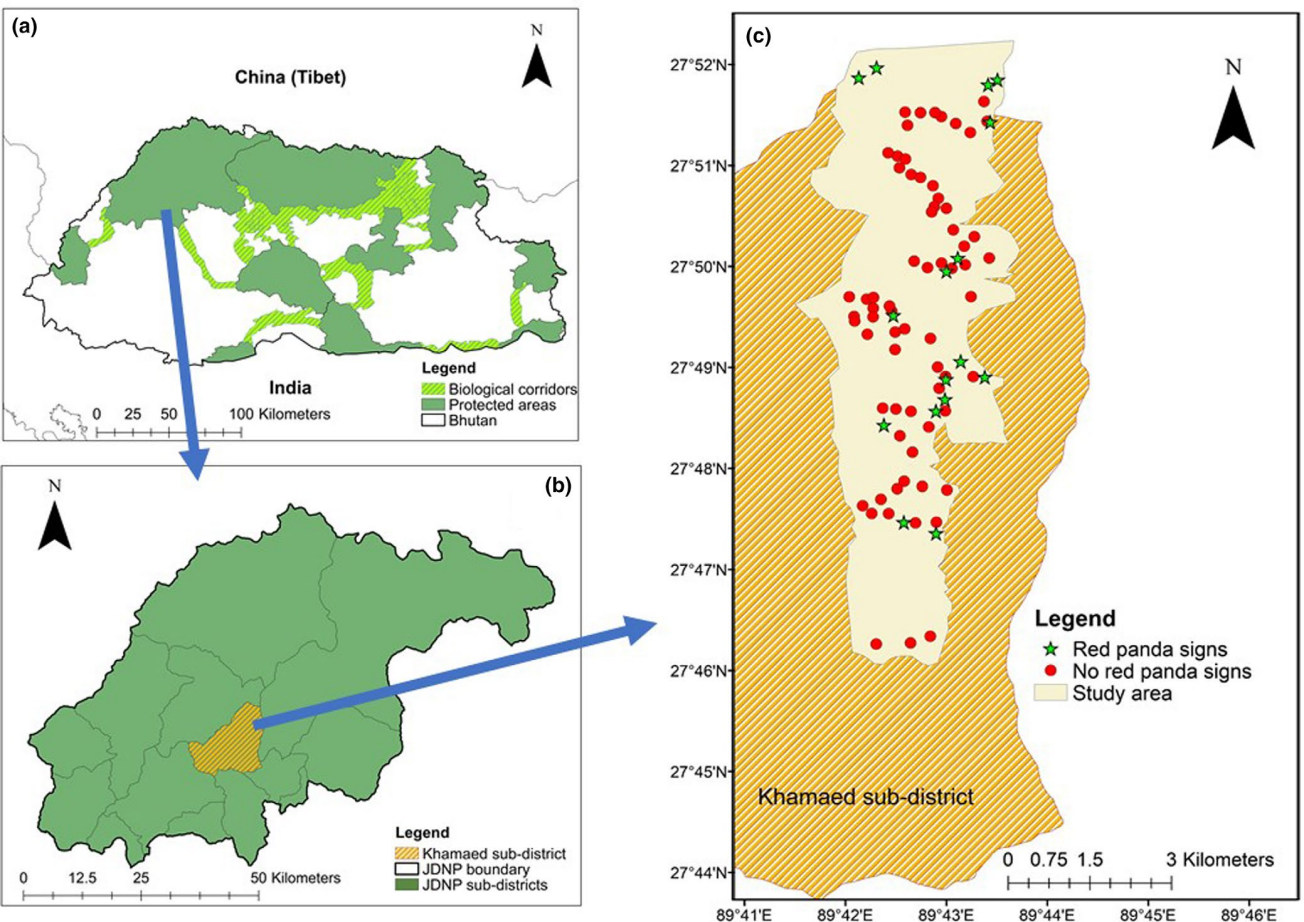
Location of the study site: (a) protected area network of Bhutan, with dark green areas representing protected areas and light green areas biological corridors, (b) Jigme Dorji National Park with its 14 subdistricts. Khamaed highlighted in yellow is one of the 14 subdistricts where the study was carried out (c) Study area with sampling plots within Khamaed subdistrict. Red and green features show the sampling plots with red panda presence and absence signs, respectively

Jigme Dorji National Park (JDNP, 27^o^33’N – 28^o^15’N and 89^o^14’E – 90^o^22’E), with an area of 4,316 km^2^, is the second largest protected area in Bhutan. It encompasses four administrative districts (Thimphu, Paro, Punakha, and Gasa) and 14 subdistricts in Bhutan (Thinley, Tharchen, & Dorji, 2015). Khamaed (27^o^43’N – 27^o^52’N and 89^o^34’E – 89^o^44’E, Figure 2b) is one of these subdistricts located in Gasa. The topography, climate, and vegetation structure within the potential red panda habitat (2000–4,500 m) is rather homogeneous across the entire JDNP (Thinley et al., 2015). This is why, we selected Khamaed subdistrict as representative of the entire national park.

### 2.2 Field survey design

We carried out the 1st phase of surveys between November 2017 and March 2018 (winter and early spring), and 2nd phase in May 2019 (spring). In the 1st phase, we surveyed 43 plots along eight trails (29 km), and in the 2nd phase, we surveyed 41 plots along six trails (21.6 km). During both surveys, we established plots and collected evidence of red panda presence, and recorded vegetation and anthropogenic disturbances.

Following Dendup et al. (2016), circular plots of 50 m radius were used to study red panda presence signs (tracks and dungs). At 500 m intervals along each trail, we established a plot in a random direction and at a random distance of 0–1,000 m (Dendup et al., 2016). Within the plot, each of the team members moved in different cardinal directions and explored for red panda signs moving toward the center of the plot and repeated this till the entire plot was scanned. If red panda signs were found in the location, we shifted the plot center to the location of the animal sign to record vegetation and anthropogenic disturbances. However, plot center was not shifted if no signs were recorded (Dendup et al., 2016). We avoided any overlap between plots.

Vegetation‐related data were recorded using tree quadrats (10 × 10 m) which were superimposed on the center of each 50 m radius plot. Understory quadrats (4 × 4 m) were superimposed at the center of each tree quadrats, and the ground cover quadrats (1 × 1 m) were superimposed on the center of each understorey quadrat (Dendup et al., 2016). Vegetation data were recorded following Schemnitz (1980) and Dendup et al. (2016, Table 1).

**Table 1:** Habitat, vegetation, and disturbance variables recorded in each different plot

Variables	Unit of measurement	Plot size	Method/Instrument used
Geographical Location	Degree Minute Second	50 m radius	GPS (Garmin eTrex Vista HCx)
Altitude	meter	50 m radius	Altimeter
Aspect	East, West, North, South, southeast, southwest, northeast, northwest	50 m radius	Suunto Compass
Slope	Degree	50 m radius	Suunto Clinometer
Poaching signs	Yes/No	50 m radius	Visual
Landslides	Yes/No	50 m radius	Visual
Livestock	Yes/No	50 m radius	Visual
Timber and NWFP harvesting	Yes/No	50 m radius	Visual
Plant disturbance (lopping/girdling)	Yes/No	50 m radius	Visual
Dead bamboos	Yes/No	50 m radius	Visual
Infrastructures	Yes/No	50 m radius	Visual
Fallen logs and stumps	Numbers	50 m radius	Visual and Count
Distance to the nearest water source	meter	50 m radius	Measuring Tape
Habitat type	Fir, CBL, Mixed conifer	50 m radius	Visual
Tree species	Numbers	10 × 10 meter	Visual and Count
Tree diameter at breast height (DBH)	cm	10 × 10 meter	Diameter Tape
Canopy cover	%	10 × 10 meter	Densitometer
Understory species	Numbers	4 × 4 m	Visual and Count
Bamboo cover	%	4 × 4 m	Visual estimation
Shrub cover	%	4 × 4 m	Visual estimation
Herb cover	%	1 × 1 m	Visual estimation

We also recorded data on disturbance variables (Table 1), including the shortest distance from the plot center to the nearest water source defined as streams or ponds (Bista et al., 2019; Dendup et al., 2016). We also recorded the number of fallen logs and stumps > 30 cm DBH (Dendup et al., 2016; Dorji et al., 2012; Sharma et al., 2014; Zhou et al., 2013).

To assess anthropogenic disturbances in red panda habitat, we recorded the presence–absence signs of plant disturbance (e.g., harvesting, lopping, girdling), livestock (sighting, droppings, hoof prints), infrastructure (power transmission lines, telecom tower, houses, roads), and poaching signs (snares). To assess natural disturbances, we recorded the presence–absence of landslides, dead bamboo, naturally fallen logs/trees (windthrow, snow damage), and carnivore signs (sighting, scats, scrapes marks, pug marks). The same team of trained foresters conducted the surveys in both phases to maintain consistency in data collection.

### 2.3 Data analysis

To calculate tree species diversity in different forest types (mixed conifer forest (MCF), cool broadleaf forest (CBL) and fir forest), following Margalef (1968), we calculated the Shannon–Wiener diversity index (H’) using the following formula:

(1) $$H^{\prime }=‐\Sigma \frac{\mathrm{ni}}{N}\;\log\;\frac{\mathrm{ni}}{N}$$

where H’ = the Shannon diversity index; ni = number of individuals of the species; and *N* = number of individuals of species.

To evaluate tree species’ dominance, following Phillips (1959), we calculated the importance value index (IVI) using the following formula:

(2) IVI=relative density+relative frequency+relative basal area

We carried out all statistical analysis in R v. 3.5.1 (R Development Core Team, 2018). Prior to performing any statistical modeling, we examined collinearity between the variables (Table 1, except Geographical Location) based on the variance inflation factor (VIF) using the usdm package (Naimi, 2015). The variables with VIF > 10 were regarded as highly correlated and omitted from further analysis (Montgomery, Peck, & Vining, 2012; Zuur, Leno, Walker, Saveliev, & Smith, 2009). Since all the habitat covariates had VIF < 2, we retained all the variables (Table 2).

**Table 2:** Results of the multicollinearity between the variables using variance inflation factor

Variables	Variance Inflation Factor
Elevation	1.52
Slope	1.31
Distance to water	1.26
Number of fallen logs	1.39
Number of understories	1.31
Ground cover	1.43
Canopy cover	1.26
Bamboo cover	1.35
Dead bamboo	1.47
Landslide	1.20
Predator	1.21
Plant disturbances	1.42
Livestock	1.45
Infrastructure	1.46

We performed logistic regression using a binomial distribution to model red panda habitat use as a function of habitat and disturbance covariates (Tollington et al., 2015). To investigate the best‐fit model, we performed multi‐model inference using a dredge function in MuMIN package (Barton, 2016). We then examined the fit of candidate models by selecting the lowest Akaike's information criterion corrected (AICc) for small sample sizes, and final model sets were restricted to ∆AICc < 1 for habitat use variables and ∆AICc < 2 for disturbance variables before model averaging (Bloker et al., 2009; Burnham & Anderson, 2002; Harrison et al., 2018).

## 3 RESULTS

### 3.1 Habitat use

Overall, evidence of red pandas was recorded in 16 of the 84 surveyed plots. In the 1st phase, seven of the 43 plots and, in the 2nd phase, nine of the 41 plots surveyed showed red panda signs.

Red panda presence within KSD was detected through 14 indirect signs in the Gayza, Dompangchung, Zomina, and Chutegompa areas, and two direct sightings in the Jabisa and Phuntshogang areas. Red panda evidence was observed between 2,414 and 3,618 m with an average elevation of 2,750.8 m (*SD* = 368.45), and the majority of the evidence was observed < 3,000 m (*n = 13*).

Interestingly, four plots with red panda signs did not have any bamboo cover. Among plots with signs of red panda, seven plots had 10%–20%, two plots had 30%–60%, and three plots had 90%–100% bamboo cover. Contrary to expectation, bamboo cover was not included in the best‐fit model (Table 3).

**Table 3:** Summary of model‐specific logistic regression (binomial distribution) models showing, degrees of freedom, AICc, ∆AICc, and model weight for habitat covariates influencing red panda habitat use in the study site

Model	*df*	AICc	∆AICc	Model weight
*CBL + Fir + Can + Und + Wat*	6	69.45	0.00	0.21
*CBL + MCF + Can + Und + Wat*	6	69.45	0.00	0.21
*Fir + MCF + Can + Und + Wat*	6	69.45	0.00	0.21
*CBL + Fir +MCF + Can + Und + Wat*	7	69.45	0.00	0.21
*Fir + Can +Und + Wat*	5	70.00	0.55	0.16

Covariates: *Wat,* distance to the nearest water source; *MCF,* mixed conifer forest**;** *Fir,* fir forest; *CBL,* cool broadleaf forest; *Und,* number of understory species; *Can,* proportion of canopy cover

Red panda habitat use decreased with distance from water sources (β = −3.15, *SE* = 1.55, 95%CI = −6.24 to −0.06), and red pandas preferred fir forest (β = 5.59, *SE* = 2.28, 95%CI = 1.05 to 10.13). Other habitat covariates such as MCF, CBL, canopy cover, and the number of understory species did not significantly influence red panda habitat use (Table 4).

**Table 4:** Summary of the model‐average coefficients and standard errors (SE) from GLM to predict covariates influencing red panda habitat use

	Estimate	SE	2.5%	97.5%
Intercept	−2.600	0.638	−3.871	−1.328
Cool broadleaf forest	−0.449	3.315	−6.967	6.068
**Fir forest**	**5.592**	**2.283**	**1.055**	**10.129**
Canopy cover	−1.440	0.757	−2.946	0.066
Number of understory species	−2.644	1.590	−5.810	0.522
**Distance to nearest water source**	**−3.152**	**1.553**	**−6.244**	**−0.060**
Mixed conifer forest	−3.961	2.927	−9.733	1.810

The significant variables, whereby the confidence intervals do not cross zero, are bolded

Red panda signs and sightings were recorded between 10 and 1,000 m distance to the nearest water sources with an average distance of 241.9 m (*SD* = 334.65). The majority of red panda records (*n = 9*) were found within 100 m from water sources.

Maximum diversity of tree species was recorded in CBL (*H’ = *4.04) followed by MCF (*H’ = *1.48) and fir forest (*H’ = *0.19). However, no association was found between red panda presence and tree diversity. Canopy cover with a range of 0%–30% showed the highest number of plots with signs of red panda presence (*n* = 12), while four of the red panda signs were recorded within plots with a canopy cover range of 60%–100%.

A total of 435 trees representing 32 species, 178 understory stems representing 30 species, and 135 ground cover representing 12 species were recorded across all plots surveyed. Of 32 tree species, only 21 species were recorded in plots with evidence of red panda with the dominance of *Tsuga dumosa* (*IVI = *58.4) followed by oak *Quercus griffithii* (*IVI = *27.7) and Nepal black cedar *Alnus nepalensis* (*IVI = *24.3). Twelve understory species were recorded in plots with red panda signs; these were dominated by *Berberis asiatica* (*n = 11*) followed by lodh tree *Symplocos* sp. (*n = 10*) and *Aconogonum molle* (*n = 6*). Six ground cover species were recorded in plots with red panda signs, but most of the plots did not have any ground cover (*n = 9*).

### 3.2 Threats

We recorded seven types of disturbances including four natural (dead bamboo, landslides, presence of carnivores, and naturally fallen logs) and three anthropogenic disturbances (plant disturbances, livestock, and infrastructures). Neither dead bamboo nor landslides were retained in the best‐fit model examining the relationship between red panda habitat use and disturbance variables (Table 5). Only plant disturbance and infrastructure significantly influenced red panda habitat use (Table 6). Although red pandas avoided areas with plant disturbance, there was a significant positive association between red panda habitat use and infrastructure presence.

**Table 5:** Summary of logistic regression (binomial distribution) models indicating degrees of freedom, AICc, ∆AICc, and model weight for disturbance covariates influencing red panda habitat use in the study site

Model	*df*	AICc	∆AICc		Model weight
*Lan + Pre +Fal + Pla +Inf*	6	81.4	0	0.337	
*Lan + Fal +Pla + Liv +Inf*	6	82.2	0.77	0.229	
*Lan + Pre +Pla + Liv +Inf*	6	82.5	1.05	0.199	
*Lan + Pre +Fal + Pla +Liv + Inf*	7	83.8	2.33	0.105	
*Lan + Pre +Fal + Liv +Inf*	6	85.2	3.8	0.05	
*Dea + Lan +Pre + Fal +Pla + Liv +Inf*	8	86.1	4.67	0.033	
*Pre + Fal +Pla + Liv +Inf*	6	86.5	5.11	0.026	
*Lan + Pre +Fal + Pla*	5	87	5.6	0.021	

Covariates: *Dea*, dead bamboo; *Fal*, fallen log; *Inf*, infrastructure; *Lan,* landslide; *Liv,* livestock; *Pla,* plant disturbance; *Pre,* predator

**Table 6:** Summary of the model‐average coefficients and standard errors (SE) from the GLM analysis of the influence of disturbance covariates on red panda habitat use

	Estimate	SE	2.50%	97.50%
(Intercept)	−1.134	0.446	−2.078	−0.305
Landslide	−17.084	1,495.325	NA	125.513
Predator	−1.048	1.201	−4.150	0.965
Fallen log	0.756	0.677	−0.619	2.082
**Plant disturbances**	**−1.410**	**0.716**	**−2.993**	**−0.098**
**Infrastructure**	**3.168**	**1.158**	**0.982**	**5.687**

Bolded are the coefficient estimates where the confidence intervals do not cross zero and the variables that are hence significant.

## 4 DISCUSSION

Evidence of red pandas in the study area was found between 2,414 and 3,618 m. Red panda presence was significantly associated with habitats close to water sources and fir forest. However, bamboo cover was not identified in the model selection as an important predictor of red panda habitat use. Red pandas avoided areas with plant disturbances, but surprisingly showed a significant positive association with infrastructure presence. Unlike plant disturbances and infrastructure, landslides, signs of predator presence, and fallen logs were not identified as important disturbance predictors of red panda habitat use. However, due to the small sample size of evidence recorded and the comparatively small area surveyed relative to the entire JDNP, further surveys will be required to confirm patterns uncovered herein. The topography of the terrain and the climate of the area made surveys in this landscape challenging and evidence of red pandas hard to detect.

A previous study conducted in the JDNP and the Phrumsengla National Park (PNP) generally recorded red pandas in CBL and conifer forests between 2,110 and 4,389 m with habitat preference for fir forests associated with bamboo undergrowth (Dorji et al., 2012). While in another separate study in the PNP, red pandas were recorded in CBL and MCF between 2,860 and 3,597 m (Dendup et al., 2016). In the present study, red panda habitat use was significantly associated with fir forests. These findings may be related to fir trees providing better nesting sites with tree cavities and being evergreen, hence providing better cover and safety (Pradhan et al., 2001; Wei et al., 1999; Williams, 2003). However, Dendup, Lham, Wangchuk, and Tshering (2018) reported preference for MCF in the forest research preserve of Ugyen Wangchuck Institute for Conservation and Environmental Research, Bhutan. This finding was reported as a seasonal habitat preference to escape colder temperatures in higher elevations during the winter, since MCF occurs in lower elevations compared to fir forests. Similarly, when the 1st phase of the current study was carried out, that is, winter and early spring, no red panda signs were observed in the fir forest. During the 2nd phase, that is, spring, fresh red panda signs were recorded in fir forest, indicating that red pandas use both higher and lower elevations during the warmer month and lower elevations in the winter.

Bamboo cover did not emerge as a predictor of red panda presence in this study, which contradicts findings from previous studies (Chakraborty et al., 2015; Dendup et al., 2018; Dorji et al., 2011; Johnson, Schaller, & Jinchu, 1988; Pradhan et al., 2001; Sharma et al., 2014). This could be attributed to the availability of other food sources, especially berries. During the 2nd phase of the study, most of the study site had a climber *Hedera nepalensis* in fruiting stage. During our study, we indeed observed traces of seeds of *Hedera nepalensis* in red panda feces.

Fallen logs, tree stumps, and distance to the nearest water source have previously been reported to positively influence red panda habitat use (Dendup et al., 2018; Dorji et al., 2012; Zhou et al., 2013). However, in the present study, except for the distance to the water source, these other covariates were nonsignificant. Proximity to water sources is evidently a critical predictor of red panda presence, further confirming findings from previous studies (Bista et al., 2017; Dendup et al., 2018; Dorji et al., 2012; Pradhan et al., 2001). Pradhan et al. (2001) reported that due to low water content in the bamboo leaves, red pandas frequently need to drink water. Bista et al. (2017) further reported that the preference for proximity to water sources may also be related to energy conservation and avoidance of predator, hence avoiding longer travel distances to access water.

Red panda presence was low in plots with plant disturbance. Red pandas generally avoid areas with plant disturbance (Acharya et al., 2018; Dendup et al., 2016). In the study site, plant disturbance was attributed to timber collection by the local people and construction of new power transmission lines. Protected areas across Bhutan allow people to live in and around its peripheries. In KSD, there are about 1,073 people (Dzongkhag Administration Gasa, 2018), and most of these people extract forest resources from the study area. Besides timber extraction, trees are clear felled for development activities especially along power transmission lines. As a right of way, a total width of 8.3 m of forest under transmission lines is cleared for 400 kilovolt (kv) transmission lines, 7.0 m for 220 kv, 6.1 m for 132 kv and 5.5 m for 66 kv (Bhutan Electricity Authority, 2008). We noticed that construction of new power transmission lines across prime red panda habitat has led to habitat fragmentation. Bhutan lost 3,460 acres of forest cover to the construction of three major hydropower projects, namely, Punatsangchu‐I, Punatsangchu‐II, and Mangdechhu, and forest cover lost as a result of hydropower projects and transmission lines ranked highest as compared to other infrastructure developments (Kuensel, 2019). Power transmission lines not only destroy wildlife habitat, but can also directly cause the mortality of individual animals, as has been recorded in langurs. Thinley et al. (2020) reported electrocution of golden langurs (*Trachypithecus geei*) in Tsirang district, Bhutan. Although the golden langur is not recorded in the JDNP, the gray langur (*Semnopithecus entellus*) is present in the national park. Red pandas being an arboreal mammal could also suffer from the presence of power transmission lines. Further studies monitoring the impact of power transmission lines on local wildlife are hence required.

Our study did not reveal a direct influence of livestock presence on red pandas; however, plant disturbance could be attributed to livestock grazing and hence reflect an indirect impact of livestock presence. For most rural Bhutanese, livestock is fundamental to their livelihood and most of livestock owners rely on forest for fodder (Wangchuk, 2002). There are about 788 cattle in KSD (Dzongkhag Administration Gasa, 2018). The majority of these domestic animals are unproductive breeds and are free ranging, and they share the red panda habitat for grazing. The competition from cattle indeed has a significant negative impact on red panda habitat use (Dendup et al., 2016; Dorji et al., 2011; Panthi, Wang, Sun, & Thapa, 2019; Sharma et al., 2014; Zhang et al., 2017).

Oddly, our study revealed that red panda presence was significantly positively associated with infrastructure presence. This could potentially reflect temporary presence or transient visits by the red panda, while moving around in search of food, new home range or mating partner on the other side of the forest fragmented by those linear infrastructures. Although we found that red panda presence was positively associated with infrastructure, we urge a cautionary interpretation of these results. More presence and absence records accounting for distance from infrastructure may be required in future studies to confirm such an unusual observation.

## 5 CONSERVATION RECOMMENDATION

Through this study, we confirmed some important habitat requirements and prominent conservation threats to red panda survival in the JDNP. Our study indicated that red panda habitat use is significantly influenced by both landscape level habitat (fir forest), microhabitat (water source), and disturbance (plant disturbances and infrastructure) variables. For any species to persist, conservationists should prioritize habitat management interventions. Plant disturbance is mainly attributed to harvesting, linear infrastructure development, and livestock. Local forest management plans should urgently be developed and implemented to regulate sustainable timber harvesting. In the case of linear infrastructure development, strict Environment Impact Assessment (EIA) should be carried out so as to avoid as much as possible impact on critical red panda habitat, especially fir forests and areas rich in bamboo. In Bhutan, forestry and environmental clearances are generally issued without taking into consideration the ecology of endangered wildlife species. In many countries, EIAs of development activities are poorly assessed and result in ineffective decision‐making and avoidance and mitigation strategies, especially when it comes to endangered species or critical habitat (Freitas, Gonclaves, Kindel, & Teixeira, 2017). There is an urgent need to recruit qualified researchers and conservation practitioners, as well as species‐level experts, especially when it comes to endangered species, to generate higher quality EIAs (Freitas et al., 2017). Globally, infrastructure development has been recognized as one of the major factors responsible for habitat loss and fragmentation, declines in wildlife populations and the introduction of invasive species (Trombulak & Frissell, 2000). Buskirk (2009) reported that medium‐ and large‐sized mammals are more sensitive to infrastructure development and are affected up to distances of several 100 meters. In the present study, we did not evaluate plot distance from transmission lines and hence explore the impact of this variable on red panda habitat use and presence; this is evidently an avenue for future research ideally suited within a pre‐ and postevaluation study of habitat use to estimate effectively the threshold distance to minimize impact on red pandas in the future.

Infrastructure development is inevitable, but negative impacts could be avoided or mitigated. Previous studies have highlighted the importance of minimizing any infrastructure development in undisturbed areas for wildlife conservation (Benitez‐Lopez, Alkemade, & Verweij, 2010). Wherever possible, power transmission lines should be constructed alongside existing roads to minimizing tree felling in undisturbed wildlife areas. Similarly, regarding livestock, in collaboration with livestock department, livestock intensification program should be initiated. Livestock intensification means keeping a smaller number of high breed cattle such as Jersey or Brown Swiss cattle. High breed cattle yield more milk (2,322 L in first lactation adjusted to 305 days) than local breeds (529 liters) and are not suitable for rugged terrains (Phangchung et al., n.d.). These cattle are usually grazed in an enclosed pasture or even if they are freely grazed, they graze along the roadsides and do not venture into high forests thereby avoiding competition with the red pandas.

## CONFLICT OF INTEREST

We declare that there is no conflict of interest that could be perceived as prejudicing the impartiality of the research reported.

## AUTHOR CONTRIBUTION

**Pema Dendup:** Conceptualization (lead); Data curation (lead); Formal analysis (equal); Funding acquisition (lead); Investigation (lead); Methodology (lead); Project administration (lead); Resources (lead); Software (equal); Validation (lead); Writing‐original draft (lead); Writing‐review & editing (equal). **Tatyana Humle:** Conceptualization (equal); Supervision (equal); Writing‐original draft (equal); Writing‐review & editing (equal). **Damber Bista:** Formal analysis (equal); Software (equal); Writing‐original draft (equal); Writing‐review & editing (equal). **Ugyen Penjor:** Formal analysis (equal); Software (equal). **Choki Lham:** Conceptualization (equal); Data curation (equal). **Jigme Gyeltshen:** Conceptualization (equal); Data curation (equal).

## Data Availability

Since red panda is an endangered species, we are not providing the data under public domain for some conservation reasons.
